# Detection of Low Frequency Multi-Drug Resistance and Novel Putative Maribavir Resistance in Immunocompromised Pediatric Patients with Cytomegalovirus

**DOI:** 10.3389/fmicb.2016.01317

**Published:** 2016-09-09

**Authors:** Charlotte J. Houldcroft, Josephine M. Bryant, Daniel P. Depledge, Ben K. Margetts, Jacob Simmonds, Stephanos Nicolaou, Helena J. Tutill, Rachel Williams, Austen J. J. Worth, Stephen D. Marks, Paul Veys, Elizabeth Whittaker, Judith Breuer

**Affiliations:** ^1^Infection, Immunity, Inflammation and Physiological Medicine, Institute of Child Health, University College LondonLondon, UK; ^2^Division of Infection and Immunity, University College LondonLondon, UK; ^3^Centre for Mathematics and Physics in the Life Sciences and Experimental Biology (CoMPLEX), University College LondonLondon, UK; ^4^Great Ormond Street Hospital for Children NHS Foundation TrustLondon, UK

**Keywords:** herpesviruses, antivirals, next-generation sequencing, immune deficiency, immune suppression

## Abstract

Human cytomegalovirus (HCMV) is a significant pathogen in immunocompromised individuals, with the potential to cause fatal pneumonitis and colitis, as well as increasing the risk of organ rejection in transplant patients. With the advent of new anti-HCMV drugs there is therefore considerable interest in using virus sequence data to monitor emerging resistance to antiviral drugs in HCMV viraemia and disease, including the identification of putative new mutations. We used target-enrichment to deep sequence HCMV DNA from 11 immunosuppressed pediatric patients receiving single or combination anti-HCMV treatment, serially sampled over 1–27 weeks. Changes in consensus sequence and resistance mutations were analyzed for three ORFs targeted by anti-HCMV drugs and the frequencies of drug resistance mutations monitored. Targeted-enriched sequencing of clinical material detected mutations occurring at frequencies of 2%. Seven patients showed no evidence of drug resistance mutations. Four patients developed drug resistance mutations a mean of 16 weeks after starting treatment. In two patients, multiple resistance mutations accumulated at frequencies of 20% or less, including putative maribavir and ganciclovir resistance mutations P522Q (UL54) and C480F (UL97). In one patient, resistance was detected 14 days earlier than by PCR. Phylogenetic analysis suggested recombination or superinfection in one patient. Deep sequencing of HCMV enriched from clinical samples excluded resistance in 7 of 11 subjects and identified resistance mutations earlier than conventional PCR-based resistance testing in 2 patients. Detection of multiple low level resistance mutations was associated with poor outcome.

## Introduction

Human cytomegalovirus (HCMV) is a ubiquitous betaherpesvirus with significant disease-causing potential in immunocompromised patients, including children with congenital immune deficiencies or immune suppression following solid organ or bone marrow transplantation. As well as causing pneumonitis, colitis, retinitis, and uveitis (Rafailidis et al., 2008) all of which contribute to HCMV-related mortality, HCMV disease also increases the risk of allograft vasculopathy and graft rejection, and significantly increases treatment costs (Hiwarkar et al., 2013). Children are at particular risk from HCMV, with over 25% of primary HCMV infections in the UK occurring in childhood (Patrick et al., 2014). Up to 16% of patients on prolonged anti-HCMV therapy develop drug resistance (Couzi et al., 2012; Shmueli et al., 2014), many of them with mutations which cause multi-drug resistance (Hantz et al., 2010). However, it may be that not all the mutations that cause resistance are known and this may lead to underestimation of drug resistance in patients failing therapy.

Three drugs are currently licensed for HCMV prophylaxis and treatment, including ganciclovir (GCV), foscarnet (FOS), cidofovir (CDV); brincidofovir (the oral derivative of cidofovir), and letermovir are in phase III clinical trials; maribavir (MBV) is available on a compassionate use basis. Treatment failure occurs in between 20 (Asberg et al., 2007) and 50% (van der Beek et al., 2012) of HCMV cases, necessitating drug changes and in some cases the use of adoptive immunotherapy. Genetic evidence of drug resistance can guide clinical decision making (Houldcroft, 2015) but current methods have technical limitations. Sanger sequencing of PCR amplicons only reliably detects drug resistance mutations that are present at frequencies of 20% or more (Sahoo et al., 2013). Deep sequencing of PCR amplicons has enabled detection of minority resistance variants at frequencies as low as 1% (Görzer et al., 2010) which could lead to earlier detection of HCMV resistance and better treatment. However, PCR and nested PCR are known to generate mutations which could make the identification of low level resistance mutations more difficult (Depledge et al., 2011). To minimize this problem, and to capture the genes currently implicated in antiviral resistance simultaneously, we made use of novel target enrichment (Depledge et al., 2011) and deep sequencing to analyse the UL27, 54, and 97 genes in serial samples from patients with prolonged HCMV viraemia despite anti-HCMV therapy. In this study, we include 11 retrospectively identified patients from Great Ormond Street Hospital for Children who had high HCMV loads for 2 weeks or longer, with clinician suspicion of anti-viral drug resistance.

## Materials and methods

### Ethics and sample collection

Whole blood samples were stored at Great Ormond Street Hospital for Children (GOSH) at −80°C. These residual samples were collected as part of the standard clinical care at GOSH, and subsequently approved for research use through the UCL Partners Infection DNA Bank by the NRES Committee London Fulham (REC reference: 12/LO/1089). All samples were anonymised. Eleven patients with HCMV viral loads that remained unchanged or rose despite 2 weeks of first line anti-HCMV therapy were selected. Twenty samples from six patients (B [5], C [2], H [4], I [4], J [4], and M [1]) were tested for UL97 and/or UL54 resistance mutations by PCR and Sanger sequencing at reference laboratories. Samples with sufficient material for DNA extraction (200 μl) were analyzed.

### DNA extraction, library construction, targeted enrichment, and sequencing

Total DNA was extracted from 200 μl each sample using the EZ1 Virus kit and EZ1 XL extraction system (Qiagen) or DNA Blood Mini kit (Qiagen) according to manufacturer's instructions. Virus loads were established by an in-house National Health Service (NHS) diagnostic qPCR assay (GOSH). To determine IU/ml, the copies/ml value is divided by 4.

### SureSelectXT target enrichment: RNA baits design

A library of 120-mer RNA baits spanning 115 GenBank HCMV whole and partial genome sequences were designed using the PATHSEEK consortium's (http://www.pathseek.eu/) in-house PERL script. Baits specificity was verified by BLASTn searches against the Human Genomic plus Transcript database. 33809 unique custom-designed HCMV baits were uploaded to SureDesign and synthesized by Agilent Technologies.

### SureSelectXT target enrichment: library preparation, hybridisation, and enrichment

Total DNA from clinical samples was quantified using the Qubit dsDNA HS assay kit (Life Technologies, Q32854) and between 200–500 ng of DNA was sheared for 150 s, using a Covaris E220 focused ultra-sonication system (PIP 175, duty factor 5, cycles per burst 200). End-repair, non-templated addition of 3′ poly A, adapter ligation, hybridisation, PCR (12 cycles pre-capture and 18 or 22 cycles post capture), and all post-reaction clean-up steps were performed according to the SureSelectXT Automated Target Enrichment for Illumina Paired-End Multiplexed Sequencing 200 ng protocol (version F.2) on the Bravo platform WorkStation B (Agilent Technologies). All recommended quality control steps were performed on the 2200 TapeStation (Agilent Technologies). Samples were sequenced using the Illumina MiSeq platform. The presence of a subset of novel SNPs was confirmed by Sanger sequencing of PCR amplicons (GATC, Germany; Source Biosciences, UK; Manchester Medical Microbiology Partnership, UK; PHE, UK; or the Royal Free Hospital Virology Department, UK).

### Sequence assembly and variant analysis

Reads were trimmed to remove adapter sequences. Total reads were mapped to the HCMV reference sequence Merlin (RefSeq ID NC_006273) ORFs UL27, 54, and 97 using CLC Genomics Workbench 8.0.3 (Qiagen). Minority variants were called if: the base was sequenced at least five times; the variant was present in at least five reads (including two forward and two reverse reads); and it was present at a frequency of at least 2% (or 1% for bases sequenced over 1000 times). The read direction filter significance was 0.05 and the relative read direction filter significance was 0.01. Variants were identified using published lists of HCMV resistance mutations (Chou, 2011, 2015a,b; Hakki et al., 2011; Göhring et al., 2015).

### Phylogenetic analysis

Consensus sequences were aligned using ClustalW (Thompson et al., 2002) and manually corrected in MEGA6 if necessary. Phylogenetic reconstructions were performed using MEGA6 maximum likelihood analysis (Tamura-Nei model, 1000 bootstraps, default settings, Tamura et al., 2013). Sequences from the following HCMV genomes were used: NC_006273.2 (Merlin), KU317610.1 (AD169), JX512198.1 (Davis), AY223527.1 (Towne), GU937742.1 (Toledo), KJ872542.1 (PAV21), HQ380895.1 (JHC), KJ361971.1 (UKNEQAS1), KJ426589.1 (Han), KP745728.1 (BE/4/2010), KP745718.1 (CZ/1/2011).

## Results

The duration of HCMV positivity and treatment for each of the 11 patients is shown in Table 1. Using SureSelect target enrichment we recovered sequence mapping to the UL27, 54, and 97 genes directly from all the clinical diagnostic samples in a single reaction without the need for virus isolation or PCR of overlapping genome fragments. A sample read mapping plot for each ORF is shown in Supplementary Figure 1. Details of mapping and coverage relative to virus genome copies/ml blood are shown in Supplementary Table 1 and Supplementary Figure 2.

**Table 1:** **Patient characteristics**.

**Patient**	**Age**	**Sex**	**No of samples**	**Underlying diagnosis**	**Peak HCMV (genome copies/ml WB)**	**Duration of viraemia (days at >10^4^gc/ml)**	**Time (days) to reduction in viraemia (<10^3^c/ml) following treatment initiation**	**Immune suppression and stimulation**	**Antivirals**	**Clinical outcomes**	**HCMV drug resistant mutations detected**	**Treated for HCMV before first sample?**
A	3 y	M	2	B-acute lymphoblastic leukemia	2134470	28	86	Ciclosporin, rituximab	FOS, GCV	Relapsed ALL after BMT; on-going treatment	None	Yes
B	2 y	F	12	Dyskeratosis congenita	65611500	200	200	NA	FOS, GCV, CDV, MBV, LEF, ART, CMV-IVIG	CMV colitis and CMV pneumonitis; death	Yes	Yes
C	1 y	M	23	Under-developed thymus (no mature T cells)	18377000	110	259	Ciclosporin, methylprednisolone sodium succinate, mycophenolate mofetil, prednisolone	FOS, GCV, ACV, palivizumab, immunoglobulin (privigen), ribavirin	Bone marrow transplant	None	Yes
D	11 y	M	3	Renal transplant	1490480	13	195	Tacrolimus, prednisolone, mycophenolate mofetil	V-GCV	Recovered	None	Yes
G	9 y	M	3	Kostmann syndrome (congenital neutropaenia)	330209	38	179	Ciclosporin, hydrocortisone, immunoglobulin (Privigen), lenograstim, methylprednisolone sodium succinate, prednisolone, rituximab, tacrolimus (topical)	FOS, GCV, CDV, ACV	Bone marrow transplant	None	Yes
H	7 m	M	5	ADA SCID	3965090	45	189	Ciclosporin, lenograstim	ACV, FOS, GCV, CDV, palivizumab	Gene therapy	Yes	No
I	11 m	M	3	DiGeorge syndrome	16721700	157	255	Ciclosporin, hydrocortisone sodium succinate, methylprednisolone sodium succinate, prednisolone	FOS, GCV, CDV, palivizumab	Thymus transplant; death	Yes	Yes
J	11 m	M	3	Acute myeloid leukemia	11728700	57	94	Ciclosporin, hydrocortisone sodium succinate, lenograstim, methylprednisolone sodium succinate	ACV, FOS	Death	None	Yes
K	12 y	M	2	Heart transplant	393192	12	218	Mycophenolate mofetil, prednisolone, tacrolimus	GCV, V-GCV	On-going treatment	None	Yes
L	16 y	M	5	Heart transplant	3091860	28	173	Mycophenolate mofetil, prednisolone, tacrolimus	ACV, GCV, V-GCV	On-going treatment	No	No
M	17 y	F	1	Chronic active EBV	256986	39	382	Ciclosporin, dexamethasone, hydrocortisone, lenograstim, methylprednisolone sodium succinate, mycophenolate mofetil, prednisolone, rituximab	ACV, GCV, FOS, oseltamivir, zanamivir	Bone marrow transplant	Yes	Yes

**Figure 1:**
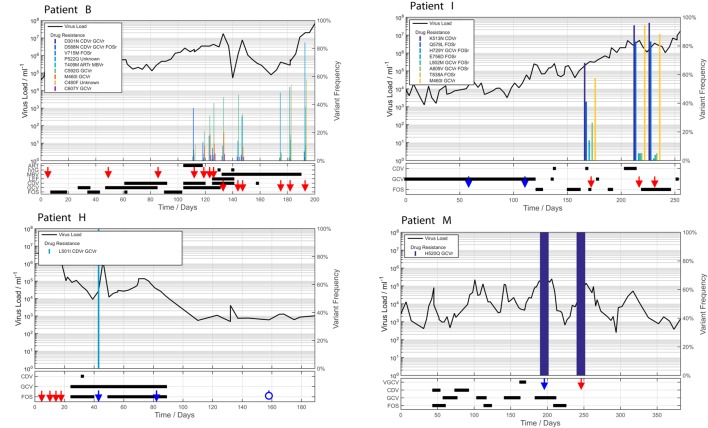
**Viraemia and anti-viral therapy in patients who developed drug resistance**. In patients B and I, multiple drug-resistance mutations accrue over time at a range of frequencies, associated with a multi-drug resistance phenotype and patient death. Patients H and M developed single fixed resistance mutations and recovered from HCMV disease. ORF locations of each mutation are Table 1. Red arrows: samples deep sequenced by target enrichment. Blue arrows: samples sequenced only by reference laboratory (PCR + Sanger sequencing) leaving insufficient material for target enriched deep sequencing. Blue open circles: bone marrow or thymus transplant, or gene therapy given. Left Y axes: log10 virus copies/ml blood. Right Y axes: variant frequency. X axis: time since admission (days).

From deep sequencing results we were able to stratify patients into two groups: those with no evidence of developing resistance mutations despite receiving long term antiviral treatment (A, C, D, G, J, K, L); and those patients who developed known HCMV resistance mutations: either fixed (H and M) or at low level (B and I). We plotted viral load, drugs received and mutations over time for each of these patients (Figure 1; Supplementary Figure 3).

### Comparison of patients with and without drug resistance mutation

Comparing the four patients who developed resistance (B, H, I, and M) versus the seven who did not, the mean duration of treatment was longer in those who developed resistance [171 (standard deviation (SD) 79) vs. 101 (SD 70) days], the median number of antiviral drugs higher (3.5 vs. 2), the peak viraemia higher (2.16 × 10^7^ vs. 5.36 × 10^6^ virus copies/ml blood) and mean duration of viraemia was greater [257 (SD 89) vs. 172 (SD 63)]. Apart from the comparison between duration of viremia (*p* = 0.048, Student's *T*-test), the other differences discussed above between the resistance and no resistance groups did not achieve statistical significance. Time to control of viremia in those who survived was faster in the two patients (H and M) with resistance: 118 days (SD 47) vs. 131 days (SD 85) in patients A, D, G, K, and L. Mean total lymphocyte counts (TLC) were persistently low in patients B, I, and J who died i.e., 0.46 (SD 0.56) as compared with patients A, D, G, H, K, L, and M who survived and controlled their viremia to below 1000 copies/ml, mean TLC 1.26 (SD 0.85).

### Patterns of resistance mutations

**Figure 2:**
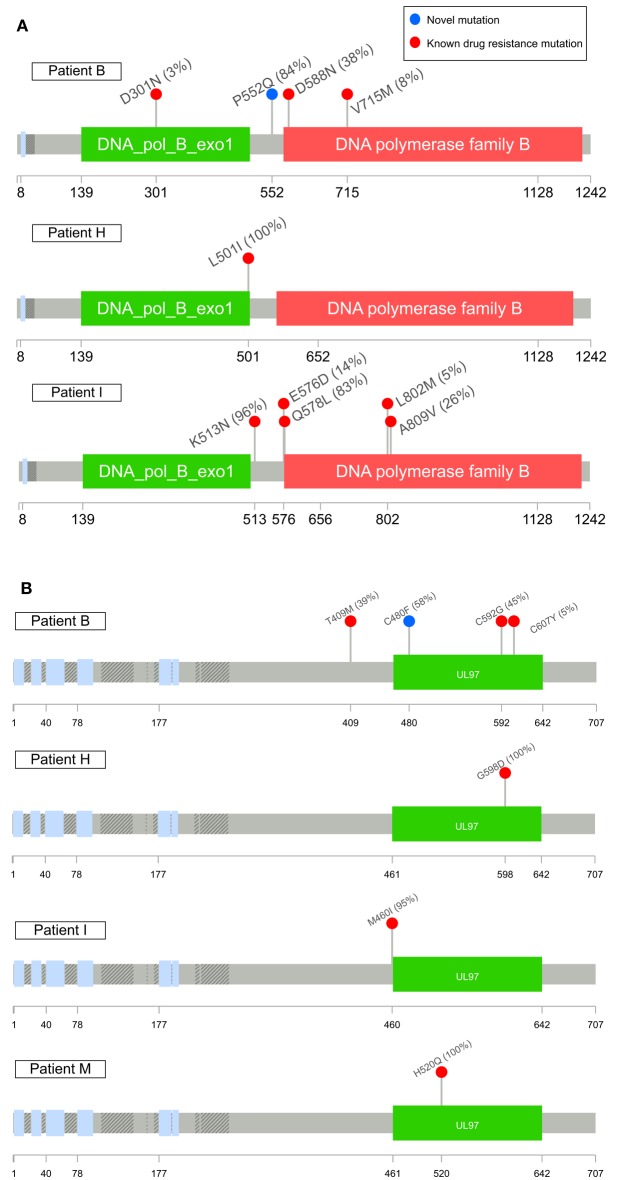
**Maps of UL54 (A) and UL97 (B) sequence variants detected in this study associated with drug resistance**. Variants in red have been previously reported as having drug resistance phenotypes or mutations with drug resistance phenotypes (G598D, Gilbert et al., 2011) when combined with concurrent UL54 mutations of unknown significance in marker transfer experiments; variants in blue are novel mutations. The highest frequency each mutation was detected at is shown in brackets. Known SNPs not shown. Plots were made using Lollipops (https://github.com/pbnjay/lollipops) and the relevant Uniprot HCMV strain Merlin protein structure.

Patients B, H, I, and M developed known drug resistance mutations in UL54 and UL97 during treatment (Figure 1; Table 2). The mean time to mutation detection was 115 days (range 18–171) following the start of antiviral treatment. The mutations detected are shown in Figure 2. Patient H carried no baseline resistance mutations by deep-sequencing analysis, but Sanger sequencing detected fixed resistance mutation L501I (CDV and GCV resistance) in ORF UL54 on day 18 of treatment (day 43 post-admission). This mutation was not detected by Sanger sequencing on day 56 of treatment despite continued GCV; the patient also received FOS throughout this period. The patient developed a mutation, G598D, in UL97 on day 81 post-admission (treatment day 56) which has previously been seen in patients failing GCV therapy, detected by Sanger sequencing. However, the phenotype of this mutation without concurrent UL54 mutations has yet to be demonstrated by marker transfer (Gilbert et al., 2011). Samples taken on days 43 and 81 post-admission were not available for follow-up deep-sequencing, but neither mutation was detected by deep sequencing on days 5, 11, 14, and 18 post-admission. No previously reported UL27 anti-viral resistance mutations were detected, although a number of SNPs of unknown function were present, reported in Supplementary Table 2, and stop codons were present in UL27 sequences from patients I and L (Supplementary Figure 4A).

**Table 2:** **All detected mutations (deep sequencing and Sanger sequencing)**.

	**UL54**		**UL97**	
**Patient**	**Known resistance**	**Novel mutations**	**Known resistance**	**Novel mutations**
A	None	D759N	None	None
B	D301N; D588N^†^; V715M	P383S; P522Q^†^; C592S; R593S; T700P	T409M; M460I; C592G; C607Y	C480F
C	None	782 frame shift; 853 frame shift	None	None
D		C988F		
G	None	C211F; E235K; E944D; S897L; D898N	None	S512STOP
H	L501I^*^	None	G598D^*^^‡^	None
I	K513N^†^; Q578L; E576D; L802M^†^; A809V	M393L; A987V	M460I	None
J	None	None	None	None
K	None	None	None	I429F
L	None		None	
M	None		H520Q^†^	

* Detected only by Sanger sequencing (sample unavailable for deep sequencing).

† Detected by deep sequencing and Sanger sequencing.

‡ Resistance phenotype unclear without concurrent UL54 mutations (Gilbert et al., 2011).

Patient M responded to FOS treatment with a reduction of viral load from ~250 k copies/ml to ~50 k copies/ml over 4 days, following the failure of GCV therapy caused by the fixed UL97 mutation H520Q known to cause an 8-fold (or greater) increase in GCV resistance (Chou et al., 2002; Göhring et al., 2015). This mutation was initially detected by reference laboratory resistance testing, and no sample remained for follow-up deep sequencing. This fixed mutation was detected 43 days after GCV therapy was withdrawn, by deep sequencing.

In contrast, patients B and I developed multiple low frequency UL54 and UL97 drug resistance mutations after 112 and 171 days of treatment respectively. Neither patient was able to control their HCMV, and both eventually died of HCMV-related complications. In patient I, the first resistance mutation at position A809V in UL54 which is associated with HCMV growth rate attenuation (Chou et al., 2007) was detected at a frequency of 26% 171 days after starting GCV. This mutation declined in frequency to ~5% following withdrawal of GCV. The cessation of GCV and start of FOS and CDV was accompanied by a rise in the GCV UL97 resistance mutation M460I to 96% together with the UL54 resistance mutations Q578L (~3-fold FOS resistance) and K513N (12-fold CDV resistance) which rose to over 80% within 46 days. This pattern suggests that the M460I GCV resistance mutation was linked on the same virus to the UL54 resistance mutations which were selected for by FOS and CDV. The rising frequency of UL54 mutations was accompanied by a rise in HCMV load from 10^4^ to 10^7^ gc/ml. Patient I died with evidence of extensively drug-resistant HCMV, carrying multiple fixed and low frequency resistance mutations to CDV, GCV, and FOS. PCR based resistance testing did not detect resistance until day 225, when only K513N was detected, although on day 171 Q578L, E756D, A809V, and M460I were at frequencies greater than 10%. One days 217 and 241, target-enriched deep sequencing detected L802M to be present at a frequency of 5%, whereas this mutation was not detected by Sanger sequencing until day 238 (68 days later), reported as a “mixture” by the reference laboratory. The reference laboratory did not report the presence of Q578L, H729Y (< 2%), E756D, A809V, T838A (< 2%) or M460I.

A similar picture emerged in Patient B. Although resistance mutations were not detectable at >2% until after day 84 following the start of treatment, multiple low frequency (< 40%) resistance mutations to GCV, FOS, and CDV, with which the patient had been treated rapidly developed thereafter (Figure 1). PCR and Sanger sequencing failed to detect these low level resistance mutations, with the exception of the GCV (D588N) substitution which was picked up Sanger sequencing 14 days after it became detectable by target enriched deep sequencing. Despite a persistently high and increasing viral load, none of the low level resistance mutations rose to fixation (peak frequency < 45%). The introduction of MBV resulted in the decline of the majority of low frequency GCV, FOS, and CDV mutations (D301N, D588N and V715M in UL54 and M460I, C592G and C607Y in UL97). In contrast T409M in UL97 rose in frequency from 2% on day 175 (43 days after commencing MBV) to 39% at the point of treatment withdrawal. Mutation T409M is known to confer cross-resistance to MBV and GCV.

### Putative novel drug resistance mutations

Potential new resistance mutations were only seen in patient B. Mutations P522Q in UL54 and C480F in UL97 were detected at days 119 (P522Q) and 175 (C480F), i.e., 14 days before and 42 days following the introduction of MBV, respectively, with the former increasing to 84% by day 193 (60 days following the start of MBV treatment) and the latter also increasing over time. P522Q and C480F have not previously been reported as resistance mutations although variants P522S and P522A are associated with GCV and CDV resistance (Chou, 2008), and C480R is associated with increased resistance to methylenecyclopropane nucleoside analogs (Komazin-Meredith et al., 2014). C480F appeared at a frequency of 5% at approximately the same time as the known MBV mutation T409M, rising to 58% by day 193 (60 days of MBV treatment). P522Q appeared first of the previously undetected mutations and rose rapidly to fixation following initiation of MBV treatment. The appearance of these three mutations was accompanied by rising viral load, suggesting that all three may confer resistance to MBV.

## Stop codons, insertions, and deletions

Patients I and L (despite never having received MBV) showed evidence of fixed truncating mutations in UL27 (Supplementary Figure 4A) both of which would be predicted to confer resistance and/or growth attenuation (Chou, 2009; Hakki et al., 2011). In patient G, a minority stop codon (~10%) was detected at amino acid position 512 in UL54 day 63 post-admission, but was not detected in subsequent samples from this patient (Supplementary Figure 4B).

In samples from a number of patients, we detected low-frequency frame shift mutations in ORF UL54, at frequencies of between 2 and 13%: A (< 5%); B (< 10%); C (< 6%); D (< 12%); H (10%); and K (13%) (Supplementary Figure 5). Many of these mutations were lost over time, or replaced by different frame shifts, suggesting they are unfit.

### Phylogenetic analysis of sequences from patients with multi-drug resistance

**Figure 3:**
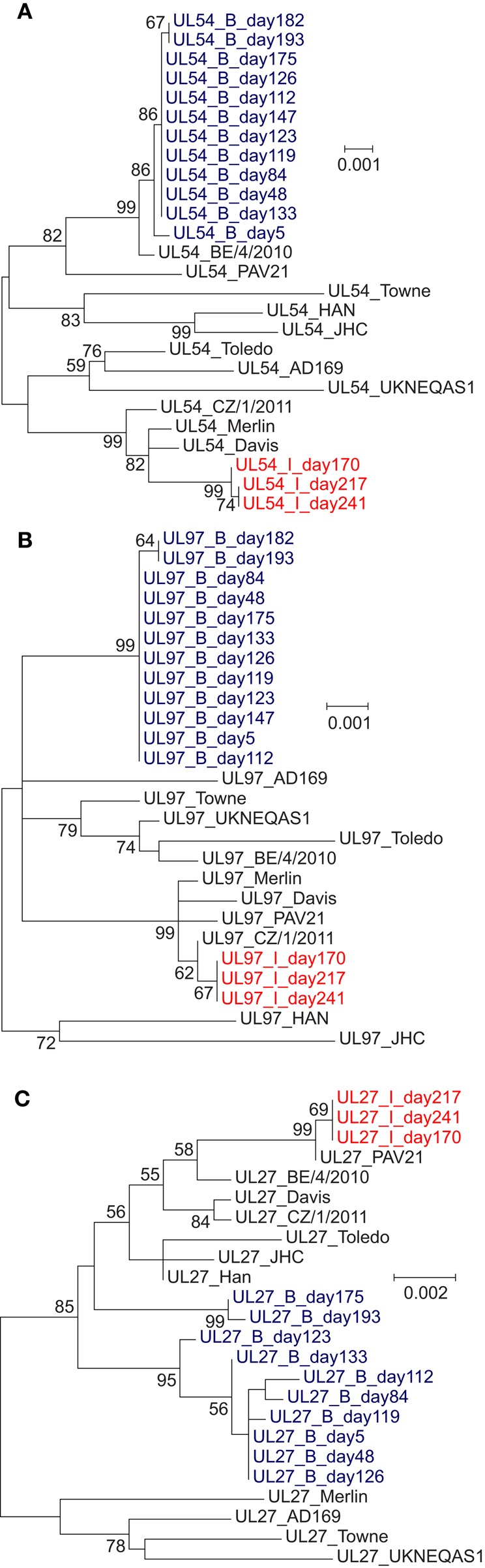
**Phylogenetic trees of (A) UL54, (B) UL97, and (C) UL27 nucleotide sequences from the patients with multiple drug-resistance mutations (B and I) and 11 laboratory and wild-type HCMV strains**. Maximum likelihood analysis was performed with MEGA6, generating a tree with 1000 replicates. Bootstrap values of 55 or above are shown. **(A)** UL54 phylogenetic tree, showing evolution of patient sequences. Changes in the consensus sequences of patients B and I due to high-frequency drug resistance mutations emerging during treatment drive the evolution of their phylogenetic position. **(B)** UL97 phylogenetic tree, showing evolution of patient sequences. Changes in the consensus sequences of patients B and I due to high-frequency drug resistance mutations emerging during treatment drive the evolution of their phylogenetic position. **(C)** UL27 phylogenetic tree. Samples from patients B do not cluster monophyletically. No previously-reported resistance mutations were detected within patient B sequences (the only patient to receive maribavir).

To examine further the complex drug resistance patterns seen in patients B and I, we constructed a phylogenetic tree for each of the three target regions, including all samples from these patients and 11 publically available HCMV genomes from GenBank (Figures 3A–C). For patient B, UL27 consensus sequences clustered in different parts of the tree in a time dependent manner (Figure 3C). The consensus sequences of genes UL54 and UL97 show change over time in patients B and I that is compatible with sequence evolution due to anti-viral drug pressure (Garrigue et al., 2016). In Patient B the changes in phylogenetic clustering for UL27 occurred after the start of MBV on day 133, and may reflect recombination or re-infection with a second strain of HCMV in this patient.

## Discussion

Persistent HCMV viraemia is associated with poor outcomes in immunosuppressed patients, including those undergoing bone marrow (Hiwarkar et al., 2013) and solid organ transplantation, and treatment with anti-HCMV drugs is indicated. HCMV viraemia carries significant economic costs, estimated at £22,500 ($32,000) per pediatric bone marrow transplant patient (Hiwarkar et al., 2013). To explore treatment failure, testing for resistance mutations and if necessary a change in therapy is recommended if the viral load remains the same or rises after 2 (Boeckh and Ljungman, 2009) or 3 (van der Beek et al., 2012) weeks of treatment. Changes in treatment may also be prompted by side effects, and bone marrow function particularly in hematological stem cell transplant recipients. In this study we used deep sequencing to investigate drug resistance patterns in persistently viremic patients requiring prolonged treatment. Notwithstanding persistent viraemia, seven patients showed no sign of drug resistance and six of them were able to control their viremia to below 10^3^ gc/ml while on treatment. Patients who developed resistance had higher viremia, lower lymphocyte counts, more drugs and longer duration of antiviral treatment, although numbers were too small for these differences to be significant. Overall, these data support the findings of others, that development of drug resistance mutations are associated with poor control of viremia and represent a poor prognostic indicator in immunosuppressed patients receiving treatment for HCMV; two of four patients developing resistance mutations died as compared with one of seven who remained resistance-free. Notwithstanding these findings, the two patients H and M, in whom resistance mutations rose rapidly to fixation, responded to a change in treatment and controlled their viremia (two qPCR results < 10^3^ gc/ml) within a mean of 17 weeks. In patient M the H520Q resistance mutation to GCV in ORF UL97 persisted despite withdrawal of the drug, suggesting that this variant remained fit despite the H520Q mutation.

By contrast, where we identified multiple mutations occurring simultaneously, in patients B and I, this was associated with profound treatment failure and death from HCMV-related disease. Observations from deep-sequencing of PCR amplicons suggest that multiple resistance mutations occurring at sub-fixation levels can contribute to a drug-resistant phenotype (Chou et al., 2014) and this is consistent with the evidence, particularly in patient B in whom high HCMV viral loads persisted in the presence of multiple often low frequency mutations (Figure 1). One explanation is that low frequency drug resistance mutations are distributed throughout the viral population resulting in many relatively unfit resistant viruses, none of which can outcompete the others (Chou et al., 2014). A similar pattern in seen in patient I, in whom a change from GCV to CDV appears to have selected for one set of resistance mutations in favor of another, perhaps because these mutations arose on different populations of the virus within this patient. Further evidence for this comes from mouse studies making use of cells infected with multiple murine CMV strains. These strains trans-complement one another, increasing overall viral fitness (Cicin-Sain et al., 2005). Both patients I and B showed a rapid rise in resistance mutations in response to treatment changes, with concomitant loss of others. This pattern, particularly in patient B for whom more samples were available, is consistent with low level persistence of multiply resistant viruses which rapidly replicate under the selective pressure of a new drug. Conventional PCR and Sanger sequencing failed on at least two occasions to detect any of these mutations, apart from the D588N which was present at a frequency of 9%. Deep sequencing is therefore able to detect potentially important drug resistance that is missed by conventional methods. For example the P552Q mutation was detected at frequencies of 1.67% (day 119), 4% (day 126), and 10.64% (day 133), prior to the start of MBV on day 133. Similarly, PCR and Sanger sequencing of samples from patient I missed multiple drug resistance mutations at frequencies of 2–41%, 54 days after these mutations became detectable by target-enriched sequencing.

The speed with which the virus became resistant in patient B and the rapid loss of four drug resistance mutations in UL97 and UL54, suggested strain replacement rather than *de novo* mutation and prompted us to examine the possibility of mixed infection. The change in phylogenetic clustering for UL27 sequences following the introduction of MBV confirmed this suspicion. HCMV is known to be highly recombigenic (Sijmons et al., 2015), and in this case, without whole genome analysis, we are unable to distinguish the possibility of recombination, re-infection, or reactivation of a pre-existing secondary strain.

In summary we have demonstrated that deep sequencing of HCMV ORFs UL27, 54, and 97 could be achieved directly from whole blood with virus loads in the range 80,000–65,000,000 copies/ml without prior culture or PCR. We were able to detect resistance mutations occurring at 2% or more in patients with viraemia persisting at levels of ≥10^4^ gc/ml for 2 weeks or more, comparable to other deep sequencing-based approaches (e.g., Görzer et al., 2010; Garrigue et al., 2016). Our data suggest that in contrast to amplicon and Sanger sequencing, our deep sequencing can exclude resistance in patients with persistently high levels of viraemia, thereby providing a measure of support prolonging current antiviral treatment or returning to them at a later date if further treatment is needed. Where resistance mutations are detected, we observed two patterns, rapid development of fixed resistance with clearance of virus following a change in treatment, and development of multiple sub-fixation resistance mutations, with potentially poorer outcome. Further investigation is needed to determine whether these patterns are indeed predictive of outcome. We do not yet understand why multiple minority drug resistance mutations arise in some patients. Multiple minority variants, which are likely to be better detected using deep sequencing methods, appeared to complicate treatment to a greater extent than single fixed resistance mutations. In our patients multiple low level drug mutations was associated with poor prognosis probably because they increased the risk that a change in drug would select for a pre-existing mutation. Deep-sequencing of HCMV allows us to characterize these mutations and could be used to inform which drugs are given earlier in treatment, or to highlight those patients for whom additional non-pharmacological interventions such as withdrawal of immunosuppression, or the use of virus-specific cytotoxic T lymphocytes are most appropriate.

## Data availability

Raw sequencing data has been deposited in the European Nucleotide Archive under project accession PRJEB12814. Bait sequences are available by request from the authors.

## Author contributions

JB, CH, and DD conceived the study design. CH, EW, JS, AW, SM, and PV supplied patient clinical data and interpretation. CH, DD, and SN performed the DNA extractions. DD, HT, CH, and SN sequenced the samples. RW administered the study. CH, JMB, and DD analyzed the data. CH, JMB, and JB wrote the paper. All authors read and approved the manuscript.

## Funding

This work was supported by funding from the European Union's Seventh Programme for research, technological development and demonstration under grant agreement no. 304875. JB is supported by the UCL/UCLH and CH by the UCL/GOSH Biomedical Resource centers. DD is supported by an MRF New Investigator Award. SN was funded by a Microbiology Society Harry Smith vacation scholarship. JB receives funding from the UCL/UCLH NIHR biomedical research center.

### Conflict of interest statement

The authors declare that the research was conducted in the absence of any commercial or financial relationships that could be construed as a potential conflict of interest.

## Abbreviations

ART: Artusenate
BCDV: Brincidofovir
CAEBV: Chronic active EBV
CDV: Cidofovir
HCMV: Human cytomegalovirus
CMV-IVIG: Cytomegalovirus intravenous immune globulin
CTL: Cytotoxic T cells
FOS: Foscarnet
GCV: Ganciclovir
GOSH: Great Ormond Street Hospital for Children, UK
LEF: Leflunomide
LTV: Letermovir
MBV: Maribavir
VGCV: Valganciclovir
WB: Whole blood.
