# Supplementary material for: Detection of Low Frequency Multi-Drug Resistance and Novel Putative Maribavir Resistance in Immunocompromised Pediatric Patients with Cytomegalovirus
Source: Front Microbiol. 2016 Sep 9;7:1317. doi: 10.3389/fmicb.2016.01317 (PMC5016526; doi:10.3389/fmicb.2016.01317)
Supplement: Supplementary file 1 [file DataSheet1.DOCX]

Supplementary figure 1: Sample coverage plots showing depth (CLC Genomics Workbench v8.5.1) of reads mapped to ORFs UL27, UL54 and UL97 in patient B, day 193. X axes: ORF nucleotide position; Y axes: mapped dead depth.

Supplementary figure 2: Plot showing HCMV copy number (copies/ml whole blood) and its relationship with average (mean) read depth across genes UL27, UL54 and UL97. Broadly, the success (as measured by mean read depth) of sequencing increases as HCMV copy number increases.

Supplementary figure 3: Viraemia and anti-viral therapy in patients who did not develop drug resistance. Red arrows: sequenced time points. Blue open circles: bone marrow, thymus or gene therapy given. Left Y axes: log10 virus copies/ml blood. Right Y axes: variant frequency. X axis: time since admission (days).

Supplementary figure 4. A: Amino acid positions of consensus-level stop codons in UL27. These patients did not receive maribavir treatment. B. Amino acid positions of minority stop variant in the UL54 sequence of patient G on day 63. Plots were made using Lollipops and the Uniprot protein structures for UL27 and UL54 respectively, HCMV strain Merlin (<https://github.com/pbnjay/lollipops>).


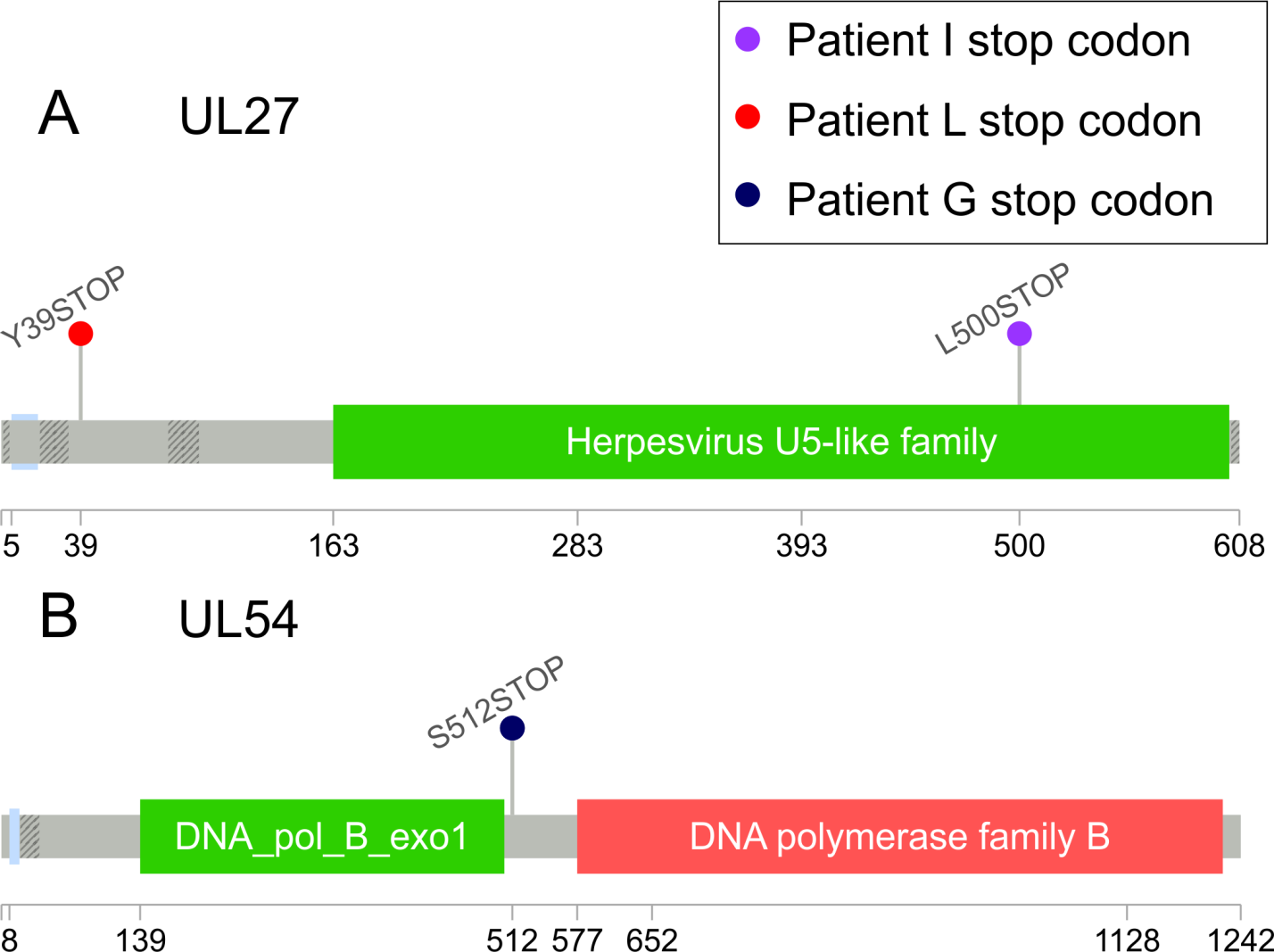


Supplementary figure 5: Amino acid positions of insertion or deletion minority variants in UL54. No indels reached frequencies greater than 13%, and many were lost at subsequent time points, suggesting these minority variants were unfit. Plots were made using Lollipops and the Uniprot protein structure for UL54, HCMV strain Merlin ([https://github.com/pbnjay/lollipops).](https://github.com/pbnjay/lollipops).#)

Supplementary table 1

Sequencing statistics (mean read depth, percentage of bases covered) for genes UL27, UL54 and UL97

| **Patient** | **Day** | **Load** | **UL27 % coverage** | **UL27 av depth** | **UL54 % coverage** | **UL54 av depth** | **UL97 % coverage** | **UL97 av depth** |
| --- | --- | --- | --- | --- | --- | --- | --- | --- |
| A | 18 | 2134470 | 100 | 86 | 100 | 273.85 | 100 | 172.81 |
| A | 28 | 335431 | 0 | 0 | 17 | 0.29 | 0 | 0 |
| B | 5 | 1106820 | 100 | 41.78 | 100 | 147.6 | 100 | 126.9 |
| B | 48 | 556941 | 100 | 54.65 | 100 | 210.09 | 100 | 138.65 |
| B | 84 | 267738 | 100 | 16.08 | 100 | 52.31 | 100 | 35.48 |
| B | 112 | 2231870 | 100 | 91.51 | 100 | 342.47 | 100 | 221.35 |
| B | 119 | 3472940 | 100 | 75.74 | 100 | 253.42 | 100 | 163.21 |
| B | 123 | 3428740 | 100 | 68.68 | 100 | 182.77 | 100 | 149.55 |
| B | 126 | 4332230 | 96 | 135.47 | 100 | 433.93 | 100 | 289.19 |
| B | 133 | 17630700 | 100 | 300.99 | 100 | 953.45 | 100 | 696.78 |
| B | 147 | 7690670 | 100 | 341.35 | 100 | 1098.86 | 100 | 704.08 |
| B | 175 | 418339 | 100 | 112.16 | 100 | 299.66 | 100 | 248.27 |
| B | 182 | 587500 | NA | NA | 100 | 136.93 | 100 | 113.74 |
| B | 193 | 11076300 | 100 | 328.22 | 100 | 948.25 | 100 | 808.35 |
| C | 1 | 18377000 | 100 | 116.46 | 100 | 580.11 | 100 | 1462.07 |
| C | 22 | 920250 | 96 | 22.62 | 100 | 349.12 | 100 | 68.35 |
| C | 103 | 106939 | 93 | 10.03 | 100 | 43.13 | 87 | 15.78 |
| D | 1 | 1490480 | 100 | 128.04 | 100 | 390.55 | 100 | 326.3 |
| D | 3 | 415366 | 100 | 124.01 | 100 | 338.92 | 100 | 254.27 |
| D | 7 | 259310 | 100 | 159.63 | 100 | 453.82 | 100 | 361.49 |
| G | 60 | 307193 | 99 | 83.12 | 100 | 327.18 | 100 | 188.98 |
| G | 63 | 330209 | 100 | 79.95 | 100 | 283.19 | 100 | 134.22 |
| G | 67 | 302256 | 100 | 49.72 | 100 | 228.91 | 100 | 94.67 |
| G | 300 | 108830 | 100 | 28.21 | 100 | 86.25 | 100 | 57.39 |
| G | 307 | 238814 | 100 | 29.31 | 100 | 78.4 | 100 | 54.38 |
| H | 5 | 3965090 | 100 | 273.07 | 100 | 1738.09 | 100 | 546.04 |
| H | 11 | 1075780 | 100 | 71.24 | 100 | 413.98 | 100 | 181.75 |
| H | 14 | 3453580 | 100 | 62.26 | 100 | 262.06 | 100 | 136.06 |
| H | 18 | 1591210 | 100 | 39.61 | 100 | 209.26 | 100 | 90.22 |
| H | 70 | 136591 | 0 | 0 | 3 | 0.03 | 0 | 0 |
| I_CMV19 | 170 | 333831 | 100 | 27.76 | 100 | 53.2 | 100 | 36.22 |
| I_CMV23 | 217 | 5114408 | 100 | 472.13 | 100 | 957.92 | 100 | 627.52 |
| I_CMV24 | 241 | 3528590 | 100 | 591.56 | 100 | 1214.41 | 100 | 748.95 |
| J_CMV20 | 21 | 988020 | 100 | 85.9 | 100 | 196.51 | 100 | 103.78 |
| J_CMV21 | 35 | 1228210 | 100 | 392.17 | 100 | 774.27 | 100 | 467.1 |
| J_CMV22 | 49 | 7189820 | 100 | 878.98 | 100 | 1883.48 | 100 | 967.96 |
| K | 106 | 393192 | 100 | 90.62 | 100 | 241.06 | 100 | 187.11 |
| K | 109 | 104165 | 100 | 30.96 | 100 | 115.55 | 100 | 67.95 |
| L | 7 | 84253 | 96 | 39.56 | 100 | 94.16 | 99 | 82.56 |
| L | 14 | 351308 | 100 | 125.9 | 100 | 358.92 | 100 | 294.34 |
| L | 21 | 3091860 | 100 | 93.48 | 100 | 590.94 | 100 | 214.41 |
| L | 26 | 564649 | 96 | 20.41 | 100 | 96.85 | 100 | 36.89 |
| M | 253 | 142524 | 100 | 11.22 | 100 | 42.09 | 100 | 39.08 |

Supplementary table 2

A number of consensus and minority variants were detected within the sequences of UL27 compared to the reference sequence Merlin. However, none of these variants has previously been associated with resistance. Only patient B received maribavir, known to induce resistance mutations in UL27 in some patients.

| **Patient** | **UL27 substitutions** |
| --- | --- |
| A | A520V |
| B | D221N, A272T, A279T, C281G/S, C283G D298S, D298G, N300S, S303N, H313Q, E318K, A332S, T342S/P, L372M, S392P, N444Y/H, C476G/S, L481M/V, R497C, A520V/P/Frameshift, P571A/T, Q588K, F598C/Y, R599Q, E602Q |
| C | None |
| D | None |
| G | A279T, C283G/S, N300S, S303N, H313Q, A332S, T342P, L372M, A435T, L436M/V, E553V/A |
| H | None |
| I | G52S, V53L, K156E, A279T, D298R, N300R, G303K, A323T, S342P, A372T, A377T, E416K, H447Y, Q500STOP, M513V, R518S |
| J | G143C, T231A, A258T, R332C, S342P, A364V, A372T, S476G, L581I |
| K | None |
| L | Q39STOP, C52S, K91N, A272T, A279T, C283G, G298S, N300S, S303N, H313Q, T342P, L372M, W377R, Q447STOP, E518K, F598C, R599Q, E602Q |
| M | None |
